# Gambling Harm and Crime Careers

**DOI:** 10.1007/s10899-016-9612-z

**Published:** 2016-04-26

**Authors:** Corinne May-Chahal, Leslie Humphreys, Alison Clifton, Brian Francis, Gerda Reith

**Affiliations:** 1 Department of Sociology, Lancaster University, Bowland North, Lancaster, LA1 4YT UK; 2 Department of Law, Lancaster University, Lancaster, UK; 3 Faculty of Arts and Social Sciences, Lancaster University, Lancaster, UK; 4 Department of Mathematics and Statistics, Lancaster University, Lancaster, UK; 5 School of Social and Political Sciences, University of Glasgow, Glasgow, UK

**Keywords:** Criminal careers, Gambling harm, Prisons, Latent class analysis, Substance use, Impulse control

## Abstract

Incarcerated populations across the world have been found to be consistently and significantly more vulnerable to problem gambling than general populations in the same countries. In an effort to gain a more specific understanding of this vulnerability the present study applied latent class analysis and criminal career theory to gambling data collected from a sample of English and Scottish, male and female prisoners (N = 1057). Theoretical links between gambling and crime were tested through three hypotheses: (1) that prisoners in the UK would have higher rates of problem gambling behaviour than the national population; (2) that if the link between gambling and crime is coincidental, gambling behaviour would be highly prevalent in an offending population, and (3) if connections between gambling behaviour and offending are co-symptomatic a mediating factor would show a strong association. The first of these was supported, the second was not supported and the third was partially supported. Latent class analysis found six gambling behaviour clusters measured by responses to the Problem Gambling Severity Index, primarily distinguished by loss chasing behaviour. Longitudinal offending data drawn from the Police National Computer database found four criminal career types, distinguished by frequency and persistence over time. A significant association was found between higher level loss chasing and high rate offending in criminal careers suggesting that impulse control may be a mediating factor for both gambling harm and criminal careers.

## Introduction

Gambling researchers across the world have observed a greater vulnerability to gambling harm amongst incarcerated offenders in comparison to men and women who gamble in general populations. A structured review of published and unpublished studies of problem gambling prevalence in prison populations retrieved 27 studies date ranged between 1977 and 2003 across Australia, Canada, New Zealand, the UK and the USA (Williams et al. 2005). Although few studies have been conducted in Europe, since 2004 further studies have been published in Canada (Turner et al. 2009, 2013), Germany (Zurhold et al. 2013), and the US (Cuadrado and Lieberman 2012). Without exception these studies of prisoners find prevalence rates that are significantly higher than those found in the relevant national population. Comparable studies in the same countries over the last 10 years obtain rates of problem gambling between 0.7 and 1.09 %, whereas prison population studies find rates of between 7.3 and 13 % (Table 1, see also Williams et al. 2005). Cuadrado and Lieberman (2012) propose that rates may be even higher (17.4 %) when arrestee populations are screened, many of whom do not proceed to prison.

**Table 1:** Summary findings of studies of problem gambling in prison populations in previous 10 years

Country	Prevalence in prison population	Scale	National rates (closest year)	Scale	% Related to offending	Sample
Canada ND (Turner et al. 2009)	13 % PG 6.3 % PG 15.7 % MR 9.4 % PG	SOGS 5+ DSM-IV-TR 5+ PGSI 3-7 PGSI 8+	2.6 % MR 0.8 % PG (2005)	CPGI	43.5 % PG 15 % MR	254 M (assessment unit)
Canada 2008-11 (Turner et al. 2013)	12.2 % F MR 12.1 % M MR 14.6 % F PG 8.2 % M PG	CPGI			44 % PG 8 % MR	381 M 41 F
Germany 2009 (Zurhold et al. 2013)	7.3 % PG (3.6 % F PG 7.5 % M PG)	Lie/Bet	0.6–1.09 % 2007, 2009	SOGS	46.7 % PG	945 M 88 F (pre-trial) 1228 M 56 F (prisoners)

Several links between gambling and crime have been proposed to explain such high levels of problem gambling in forensic populations (Arthur et al. 2014; Campbell and Marshall 2007; Williams et al. 2005). In summary, three connections have been identified; an ‘instrumental link’ where crime facilitates gambling (such as stealing to pay off gambling debt), a ‘co-symptomatic’ relationship whereby a mediating factor may increase the probability of both offending and gambling (such as alcohol or substance use) and, finally, a ‘coincidental’ connection where crime and gambling are not directly related but merely participated in by the same people (Lahn and Grabosky 2003, p. 19). Some previous research that has focused on the types of crimes committed by people classified as problem gamblers (see for example Bellringer et al. 2009; Laursen et al. 2016), finds that although there is a higher likelihood of problem gamblers being charged with an offence, there is no association between the type of offence and gambling. One approach that has not been explored is the potential link between gambling harm and crime careers. Criminal career research (Blumstein and Cohen 1979; Farrington 1995; Macleod et al. 2012; Soothill et al. 2009) examines patterns of offending over extended periods of time and therefore offers an opportunity to explore career patterns that may be related through co-symptomatic and/or coincidental connections to both offending and gambling behaviour.

## Overview of the Present Research

Building from this research, the present study aimed to test three hypotheses; firstly that offenders in the UK would have higher rates of problem gambling behaviour than the national population. Secondly, that if gambling and crime were coincidental, gambling behaviour would be highly prevalent in an offending population. Thirdly, if connections between gambling behaviour and offending are co-symptomatic a mediating factor such as substance use would show a strong association. These hypotheses were tested through two data sets; cross sectional gambling prevalence data from a sample of offenders in the UK and longitudinal offending data for a subset of this cohort. Results of a descriptive analysis of gambling prevalence data confirm the first hypothesis but not the second. The third hypothesis was tested through analysis of descriptive bivariate statistics relating to gambling behaviour—identified according to Latent class analysis—and drug use. This was partially supported. Six latent class gambling groups are identified, primarily differentiated through loss chasing behaviour and its consequences. Four criminal career types distinguished by frequency and persistence of offending across the life-course were identified. Gambling harm and crime careers were found to be co-symptomatic with alcohol use and also with high frequency offending. Findings are considered in the context of theoretical frameworks for distinguishing sub-types of gambler; specifically the pathways model (Blaszczynski and Nower 2002), and a ‘co-symptomatic’ relationship between gambling, crime careers and impulse control is proposed. The analysis is situated within the context of a public health approach to gambling that promotes targeted and tailored help for populations who may be vulnerable to gambling harm (Korn and Shaffer 1999). The results offer a nuanced categorisation of gambling harm that indicates the need for different levels of targeted interventions within offending populations.

## Measuring Problem Gambling

Gambling prevalence studies report on prevalence rates as measured by various problem gambling measurement instruments, referred to as problem gambling screens or scales. The scales include a range of items, many of which are held in common, the majority derived from the Diagnostic and Statistics Manual 4th Edition (DSM-IV) (American Psychiatric Association 1994). Until recently problem gambling was classified as an impulse control disorder not elsewhere classified, and although in the latest edition (DSM 5) it is categorised as a behavioural addiction, the criteria for gambling disorder remain similar (American Psychiatric Association 2013). All the scales have some items that measure an element of impulse control. Most commonly questions focus on whether respondents; gamble longer, with more money or more frequently than intended, chase losses (trying to win back money lost), and/or make unsuccessful attempts to cut down, stop or control their gambling (Williams and Volberg 2010). Scales vary in relation to other elements measured including an emphasis on financial aspects (South Oaks Gambling Screen (Lesieur and Blume 1987) and greater emphasis on the health and psychological consequences of gambling, such as the Canadian Problem Gambling Index (Ferris and Wynne 2001) and the Victorian Gambling Screen (Ben-Tovim et al. 2001). Given the range of possible responses to such varied categories, rather than forming homogenous ‘risk’/problem groups, it is possible that respondents can appear similar but for quite different reasons. For example, someone who agrees that they frequently chase their losses and also frequently spends more money than intended would score sufficiently highly to place them in the problem gambling category on some scales, indicating that their gambling may be theoretically addressed as an impulse control disorder. However, a respondent could also score in the problem gambling category by agreeing that they frequently lied about their gambling behaviour to others and/or they have frequently gambled to escape from pressures of everyday life, indicating quite different motives and consequences that require alternative theoretical explanations (Blaszczynski and Nower 2002).

LCA (see method) allows for testing whether different subtypes of gambling may exist in the population of interest. Gamblers who rate as low, medium risk or problem gambler on the various measures may be similar in terms of the *frequency* of problems they experience but different in terms of the *type* of problem and to the extent that they recognise gambling harm, which would impact on their motivation to take up treatment. Understanding differences between gambling groups may be particularly important within criminal justice systems when considering the allocation of scarce resources to prevent future recidivism. It may be that prisoners can be offered interventions that are more sensitive to gambling behaviour type aimed at reducing both harmful gambling behaviour and criminal career.

## Method

### Measures

Gambling behaviour was measured using the Problem Gambling Severity Index (PGSI), developed specifically for application to cross-sectional population studies (Wynne 2003). The PGSI is comprised of nine scored items, four that reference harmful consequences and five that reference gambling behaviours. Using this scale gamblers are placed in one of four gambler categories depending on a score that can range from 0 to 27: non-problem gamblers (0 score), low risk (score 1–2), moderate risk/problem gamblers (score 3–7) and severe problem gamblers (score >8). The PGSI was selected as the measure of gambling behaviour for the present study for three reasons; firstly because it provided proxy measures for the consequences (or harms) of gambling; secondly, because it offered the opportunity to compare national population data using the same screen at a similar point in time (Wardle et al. 2011); and thirdly, secondary analysis of large population samples in Canada reveals that the PGSI discriminates well between the severe problem gambler and non-problem gamblers and that both groups have validity as distinct subtypes (Currie et al. 2012). Respondents were asked to rate their replies to the nine PGSI items for the 12 months prior to entering prison.

In addition a number of variables measured substance use (including alcohol). These assessed type of substance used in detail and frequency of use in the 12 months prior to incarceration. The question ‘Have you received help for your substance use issues?’ and a question asking about frequency of substance use were combined to create a single variable (type of drug user) with four categories:

- Daily user not received help for substance use
- Weekly/monthly user not received help for substance use
- Daily user received help for substance use
- Weekly/monthly user received help

Longitudinal offending data were downloaded from the UK Police National Computer, which contains criminal histories drawn from operational records. In addition to confirmed offences for England, Wales and Scotland it also includes all recorded cautions and warnings.

### Participants and Procedure

A signed consent form was requested for completion of the questionnaire and also access to past (lifetime) and future (up to 18 months following questionnaire completion) offending records through the PNC. Ethical approval was obtained through the Integrated Research Application System, the Ministry of Justice and the Prisons Research Committee. The questionnaire was administered across prison sites in England and Scotland. 1200 questionnaires were distributed equally across six sites; three prisons in England (two male and one female), two male prisons and one female electronic monitored site in Scotland. Prisons were selected on the basis of their regional distribution (North, South), sex (male, female) and Category (C or D, meaning that prisoners were classified as at low risk of escape). Sampling within prisons was necessarily opportunistic. Following meetings with the Senior Management Team in each site a Prison Project Co-ordinator (PPC) was appointed from within the prison staff establishment. The rollout was achieved over a full regime day. PPCs generally outlined a ‘rollout plan’ detailing where to best distribute questionnaires at specific times. The PPC harnessed staff and 2–4 prisoner volunteers across wings and billets to help administer the survey during these times including; meal times, lock down (i.e. those left unlocked doing jobs), queuing for canteen food at tea time, during the working day in employment (workshops/kitchens), those in education, prisoners on wings or in billets (unemployed), and in library sessions. 1057 questionnaires were returned across the six sites giving an overall response rate of 88 % [546 (51.7 %) in England and 511 (48.3 %) in Scotland].

252 women and 805 men completed the questionnaire giving a total distribution of 24 % female and 76 % male. Although women comprised approximately 5–6 % of the general prison population at the time our survey was conducted, they were deliberately over sampled to enable meaningful statistical analyses of sex differences. The mean age of the sample was 33.5 (32.2 in Scotland (range 20–64, SD 8.9), 34.7 in England (range 18–77, SD 11.3). This was broadly comparable with the main prison population at the time (Ministry of Justice 2010). In terms of ethnicity, 87.5 % were white/white British, 4.9 % were Asian/Asian British and 4.6 % were Black/African/Caribbean/Black British with less than one percent falling into other categories. The Scottish sample was ethnically representative of prison populations in Scotland, however, the English sample had a higher percentage of white participants (82 vs. 72 % in English prisons).

## Analysis

Odds ratios were calculated to test for differences between prevalence of gambling problems according to standard PGSI gambling types. 95 % confidence intervals are calculated for these ratios. For the analysis that determined to identify if more nuanced gambling types exist Latent Class Analysis was used. LCA (Vermunt and Magidson 2003), a statistical method for finding subtypes of related cases (latent classes), was applied to the responses to the PGSI items. LCA allowed for each respondent to be probabilistically assigned to a cluster (or sub-type) according to their responses to questions concerning their gambling behaviour. It is analogous to cluster analysis in that the aim of both methods is to find hidden or latent classes in a dataset. The main difference is that LCA is a statistical method (rather than mathematical) that takes account of the fact that cases assigned to a particular cluster are similar but not identical. LCA software produces a profile that characterises each cluster. For example, the profile of a cluster may be that cases are *very likely* to answer ‘Almost Always’ to questions one, three, and five and ‘Never’ to all other questions.

LCA can be used where cases are characterised by multivariate categorical variables. A number of studies exist that have used LCA on different types of data. For example, Deslauriers-Varin and Beauregard (2010) used data relating to the target selection process of sex offenders, Vaughn et al. (2009) used criminal history information, and Jackson and Kuha (2014) used information about participants’ fear of crime. In all instances the analysis identifies clusters that are similar in terms of the repertoire of responses to the items of interest. With regard to the research presented in this paper a case is an individual’s set of responses to the PGSI and each response is a categorical variable with four possible outcomes: 0 = never, 1 = sometimes, 2 = most of the time, 3 = almost always. Our interest was to identify offenders who are similar in terms of their responses to the PGSI taking account of each of the 9 items and all the possible outcomes.

All respondents were included in the gambling behaviour LCA analysis regardless of whether they had no problems at all, showed problematic behaviour in terms of all PGSI questions, or were somewhere in between. This is because it is possible that a group may be found that suggests that, statistically, there are no significant differences between offenders who do not experience any harm and those who experience very little. On the other hand, the best model may show that offenders who have no gambling problems are mutually exclusive to all other groups.

‘Don’t know’ responses were treated as missing and valid responses were treated as ordinal. A small proportion (6 %) of respondents either did not answer or responded with ‘don’t know’ on at least one of the nine PGSI questions. However, in order to maximise the information that was retrieved these cases were included in the analysis as *Latent GOLD* ^**®**^ provides valid estimates where there is missing data. Of the 1057 prisoners who completed the survey 1056 were included in the initial LCA analysis (only one prisoner responded ‘Don’t Know’ to all questions).

In order to improve statistical analysis the English and Scottish PGSI responses were merged when undertaking LCA. The PGSI items were:

- Gone back to try to win money lost
- Bet more than could afford to lose
- Borrowed money or sold anything to get money to gamble
- Gambled and spent more to get the same amount of excitement
- Gambling has caused financial problems
- Felt that might have a problem with gambling
- People have criticized betting or told that have gambling problem whether think it is true or not
- Felt guilty about the way gamble or what happens when gambling
- Felt that gambling has caused health problems, including stress and anxiety

Table 2 provides the correlation matrix for these variables.

**Table 2:** Correlation matrix for PGSI variables

		1	2	3	4	5	6	7	8	9
1. Gambled when could not afford to loose	Pearson correlation	1								
	*p*									
2. Gambled more to get same feeling of excitement	Pearson correlation	.634	1							
	*p*	.000								
3. Gambled to win back losses	Pearson correlation	.701	.616	1						
	*p*	.000	.000							
4. Borrowed/sold to gamble	Pearson correlation	.678	.723	.620	1					
	*p*	.000	.000	.000						
5. Have problem with gambling	Pearson correlation	.603	.627	.592	.708	1				
	*p*	.000	.000	.000	.000					
6. Gambling caused health problems	Pearson correlation	.492	.451	.444	.533	.619	1			
	*p*	.000	.000	.000	.000	.000				
7. Been criticized for gambling	Pearson correlation	.575	.597	.575	.645	.780	.646	1		
	*p*	.000	.000	.000	.000	.000	.000			
8. Gambling caused financial problems for family	Pearson correlation	.591	.574	.544	.690	.725	.707	.731	1	
	*p*	.000	.000	.000	.000	.000	.000	.000		
9. Felt guilty	Pearson correlation	.555	.591	.537	.646	.723	.624	.703	.704	1
	*p*	.000	.000	.000	.000	.000	.000	.000	.000	

Criminal careers were analysed where respondents gave permission. Of the total (1057), 757 (72 %) provided consent to access their offence data from the PNC database. LCA was applied to data on offending frequency and age to identify crime career clusters for this group, which then allowed the analysis to test for any significant relationships between crime careers and gambling harm.

## Results

In line with previous studies, and in support of the first hypothesis, the overall prevalence of problem gambling in the prison sample as measured by the PGSI was significantly higher than that found in the general population for the same time period (12.1 % compared with 0.7 % (Wardle et al. 2011) see Table 3). The second hypothesis, that if gambling and crime had a coincidental relationship gambling participation would be higher in a prison population, was not proved. The prevalence of gambling behaviour was significantly lower in this population; a larger percentage of prisoners have not gambled in the year prior to prison, compared with the national sample (42.7 vs. 26.9 %; *p* < .001). However, where prisoners did gamble, a significantly lower percentage gambled without problems (23.0 vs. 64.9 % of the national population; *p* < .001). Differences in non- gambling and non-problem gambling levels between Scotland (33.3, 29.0 %) and England (51.5, 17.4 %) were also significant (*p* < .001) (see Table 4). In England, overall a lower proportion of prisoners participated in gambling than in Scotland (48.5 vs. 66.7 %), and in the Scottish sample a higher percentage appeared to gamble without problems (29 vs. 17.4 %). There were no significant differences between the two countries for those who scored 3–7 and >8; with 10.4 % (England) and 13.9 % (Scotland) reaching the PGSI problem gambling threshold.

**Table 3:** Problem Gambling Severity Index Scores for prisoners in Scotland and England compared with UK population 2010 (BGPS)

Gambling Score	Total prison sample		BGPS (2010)		Odds ratio (prison sample/BGPS)	95 % confidence interval	
	N	%	N	%		Lower	Upper
Non-gambler	451	42.7	2084	26.9	2.0	1.8	2.3
Non-problem gambler (0)	243	23.0	5028	64.9	.2	.1	.2
Low threshold (1–2)	118	11.2	434	5.6	2.1	1.7	2.6
Medium threshold (3–7)	116	11.0	147	1.9	6.4	5.0	8.2
Problem threshold (8+)	128	12.1	54	0.7	19.7	14.2	27.2
Total	1056	100.0	7747	100.0

**Table 4:** Problem Gambling Severity Index Scores for prisoners in Scotland compared with prisoners in England

Gambling Score	Scotland		England		Odds ratio (Scotland/England)	95 % confidence Interval	
	N	%	N	%		Lower	Upper
Non-gambler	170	33.3	281	51.5	.5	.4	.6
Non-problem gambler (0)	148	29.0	95	17.4	1.9	1.4	2.6
Low threshold (1–2)	55	10.8	63	11.5	.9	.6	1.4
Medium threshold (3–7)	66	12.9	50	9.2	1.5	.9	2.2
Problem threshold (8+)	71	13.9	57	10.4	1.4	.9	2.0
Total	510	100.0	546	100.0			

PGSI scores were also analysed in relation to age, sex and ethnicity. No significant association was found between age or ethnicity and gambling behaviour in either country sample. There were significant sex differences, however, with female offenders 1.6 times less likely to gamble than their male counterparts (*p* = *0.001*).

### Latent Class Analysis Gambling Behaviour Clusters

**Fig. 1:**
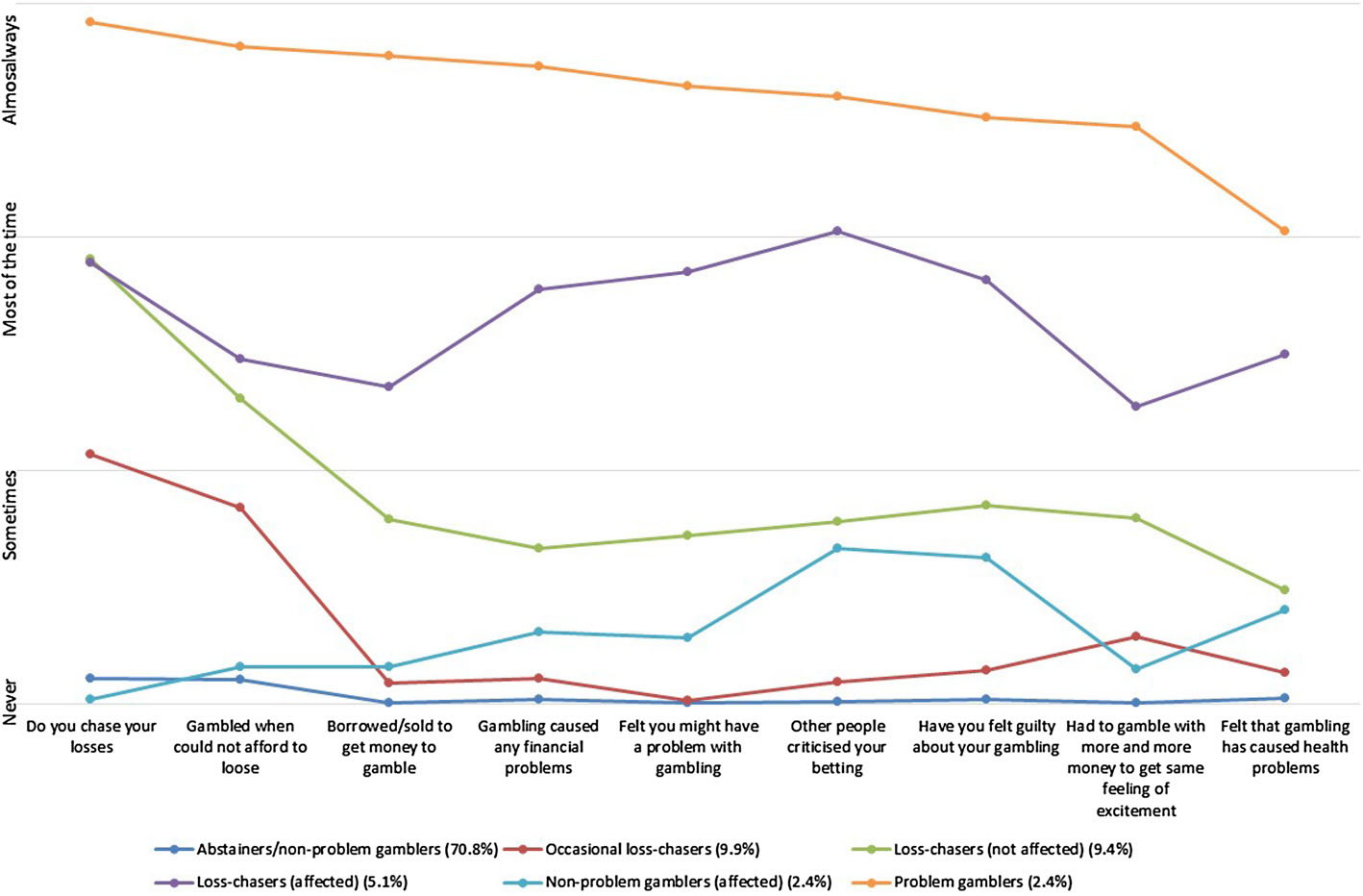
Plot of mean scores of each of the Problem Gambling Severity Index items for each latent class

Six latent class clusters emerged within this combined English/Scottish prison’s data set. The six clusters found through the LCA are outlined below and the elements on which they clustered are depicted in Fig. 1:

#### Cluster 1: Abstainers/Non-problem Gamblers (N = 750)

This was the largest cluster comprising 71.0 % of the total sample. The majority of members of this group claimed not to have gambled in the 12 months prior to entry to prison (N = 692). Members of this group that did gamble (N = 58) were very unlikely to agree that they experienced behaviours associated with problem gambling such as chasing losses and borrowing money to gamble or that they experienced any adverse consequences, such as feeling guilty or associated health problems.

#### Cluster 2: Occasional Excitement Chasers, Claiming to be Rarely Affected (N = 111)

The second largest group, 10.5 % of the sample, clustered into what could be described as ‘occasional excitement chasers’. Individuals in this group agreed they occasionally spent more to get the same feeling of excitement and chased the losses they made as a result of gambling. In addition, they sometimes gambled when they could not afford to. However, they did not borrow money to fund their gambling and they were not likely to be affected by other adverse consequences of their gambling as measured by the PGSI. By way of comparison with the more standard screening measures, members of this group scored as low risk (N = 51) or medium risk (N = 58) on the PGSI (see Tables 3, 4).

#### Cluster 3: Occasional Loss Chasers Agreeing They Were Sometimes Affected by Adverse Consequences N = 98

A slightly smaller group (9.3 % of the total) admitted to chasing losses more frequently than cluster two and gambled when they could not afford to on a more frequent basis than cluster two. They were not very likely to borrow money for gambling. If they were affected by the adverse consequences of their gambling they agreed this was only occasionally. However, under the PGSI screen, 55 of this group met the threshold for problem gambling and 43 for medium risk.

#### Cluster 4: Occasional Loss-Chasers More Seriously Affected by Adverse Consequences (N = 52)

All members of cluster four met the PGSI problem gambling threshold and comprised 5 % of the total sample. In the LCA they were characterised by their occasional loss chasing and experience of higher-level effects as a consequence of their gambling behaviour. Individuals in this cluster were very similar to cluster three in terms of the frequency with which they displayed loss-chasing behaviour. However, they were all likely to be affected at least sometimes by the adverse consequences of gambling. Most agreed they had a problem with gambling and were frequently criticised for it.

#### Cluster 5: Unassertive Gamblers (N = 24)

2.2 % met the criteria for either low (N = 9) or moderate risk (N = 15) on the PGSI who might be described as ‘unassertive gamblers’. Members of this group rarely, if ever, chased losses nor did they pursue excitement and appeared to gamble with few problems. However, they were more likely to be occasionally criticised and to occasionally feel guilty about their gambling behaviour.

#### Cluster 6: Frequent Loss Chasers Seriously Affected by Adverse Consequences (N = 21)

2 % of the sample were frequent loss chasers who were also likely to experience all the adverse consequences of excessive gambling. All members of this group met the PGSI problem gambling threshold.

Figure 1 provides a graphical summary of the clusters. Higher scores on the Y-axis represent greater frequency with which the behaviours/consequences are experienced as measured by the PGSI (X-axis). Responses to each item are on a 4-point Likert scale and the location of each marker for each cluster represents the average Likert scale score for that item. For ease of interpretation the X-axis contains the qualitative labels for the Likert scale.

Thus, approximately one third of the total (29.0 %) agreed they were experiencing some harms associated with their gambling ranging from infrequent to severe. Three of the LCA groups were distinguished by their loss chasing behaviour; a quarter of the total sample (24.7 %) fell into one of these groups (see Table 5). For the majority of these, harmful consequences of their loss chasing appeared to be less frequent. A group of ‘higher level’ occasional loss chasers (4.9 %) were likely to be more negatively affected by their gambling behaviour with 2 % of the total sample being those endorsing *all* PGSI items as ‘most of the time’ or ‘almost always’ (see Table 5).

**Table 5:** Gambling behaviour types derived from latent class analysis

Latent gambling group	N (N used for calculation of mean PGSI scores^a^)	% of total sample	Mean PGSI score (SD)	Most common types of conviction that led to imprisonment	
Abstainers/non-problem gamblers	750 (198)	71.0	.3 (.5)	Violence/firearms/weapons	29.5 %
				Possession/supply/importation of drugs	23.4 %
				Theft	17.1 %
Occasional excitement chasers	111 (90)	10.5	2.8 (.9)	Violence/firearms/weapons	28.8 %
				Possession/supply/importation of drugs	28.8 %
				Theft	16.4 %
Occasional loss-chasers (higher-level less affected)	98 (68)	9.3	8.3 (2.0)	Violence/firearms/weapons	31.3 %
				Other/public order	20.3 %
				Theft	18.8 %
Occasional loss-chasers (higher level affected)	52 (41)	4.9	15.5 (2.3)	Violence/firearms/weapons	25.0 %
				Theft	15.6 %
				Possession/supply/importation of drugs	15.6 %
Unassertive gamblers	24 (20)	2.3	3.2 (1.2)	Theft	29.4 %
				Violence/firearms/weapons	23.5 %
				Burglary	11.8 %
Gamblers affected by high level of problems	21 (20)	2.0	23.8 (2.8)	Possession/supply/importation of drugs	26.7 %
				Theft	20.0 %
				Fraud and forgery	20.0 %
Total	1056 (437)	100			

^a^A small proportion of respondents either did not answer or responded with ‘Don’t know’ on at least one of the PGSI questions. For Tables 3 and 4 only offenders who gave valid responses to all nine questions are included in the mean PGSI score calculations. Furthermore, Cluster 1 contains 451 abstainers and these offenders did not complete the PGSI questions. Hence the 198 in this table are non-problem gamblers who completed *all* PGSI questions

Table 5 also provides an insight into the type of offending behaviour that led to imprisonment for the offenders in each group. The crime types that most commonly led to imprisonment are listed. Whilst numbers are small it is of note that the most seriously affected gamblers are the only ones to include fraud and forgery amongst their offences and to not include violent offences in their criminal careers.

This model offers a more heterogeneous picture than that given when the PGSI global risk categories are used (see Table 6). Of particular interest is the distribution of the ‘problem gambler’ group across the different LCA sub-types. Under the PGSI scoring, problem gamblers included 12.4 % (N = 128) of the sample. 16 % of these (2 % of the total sample, N = 21) agreed they experienced the most severe consequences of their gambling behaviour across a range of items measuring impaired control, financial harm, health problems, self-identifying as problem gamblers and reporting high levels of criticism from others. 41 % of prisoners scoring as problem gamblers on the PGSI (N = 52) cluster as occasional loss chasers who are likely to be affected, at least sometimes, by the adverse consequences of gambling. This cluster (cluster 4) also self identify as problem gamblers but the motivation of excitement appears less likely for this group. The remaining 43 % of PGSI problem gamblers (5 % of the total, N = 55) join cluster three as occasional loss chasers who are statistically less likely to report negative effects. At the other end of the scale PGSI ‘low risk’ gamblers appear to either gamble without any negative effect (N = 58) or agree they occasionally chase losses but do not seem to experience the harms associated with loss chasing apparent in other clusters. The majority of PGSI ‘medium risk’ gamblers also agree they are occasional loss chasers who report experiencing negative effects infrequently or sometimes (see Clusters 2 and 3 above).

**Table 6:** Comparison of PGSI categories to latent gambling sub-types

Latent gambling group	PGSI risk categories (PGSI Score)				Total	%
	Non-PG (0)	Low risk (1–2)	Moderate risk (3–7)	Problem gamblers (8 +)		
Abstainers/non-PG	692	58	0	0	750	71.0
Occasional excitement chasers	2	51	58	0	111	10.5
Occasional loss-chasers (higher-level sometimes affected)	0	0	43	55	98	9.3
Occasional loss-chasers (higher level affected)	0	0	0	52	52	4.9
Unassertive gamblers	0	9	15	0	24	2.3
Problem gamblers	0	0	0	21	21	2.0
Total	694	118	116	128	1056	100

### Gambling Behaviour Clusters and Criminal Careers

**Fig. 2:**
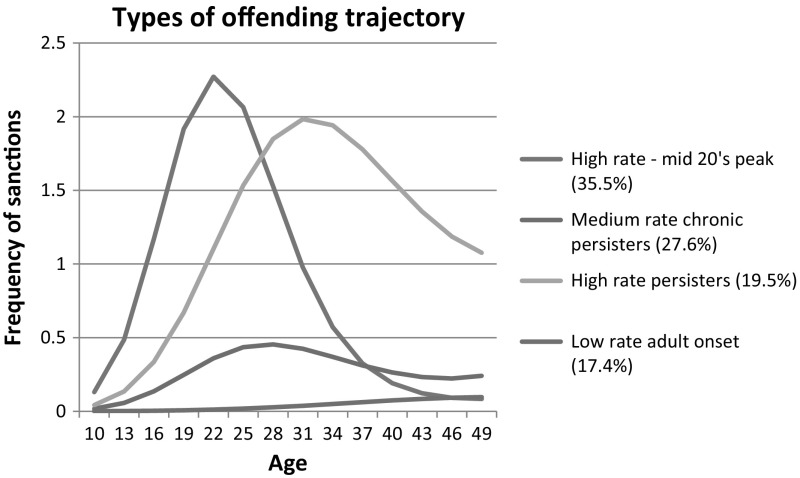
Types of offending trajectories for the prison gambling sample in England and Scotland (N = 757)

LCA was applied to the offending histories of those who gave consent to PNC database access (N = 757). Frequency of offending, length of criminal career, and age were included in the LCA. This enabled analysis of criminal career types and testing for any statistical association between gambling behaviour and crime careers. Four criminal career clusters were identified that grouped respondents according to their probability of offending (offending rate) and time of offending (age of onset, age at offence). The clusters were: high rate offenders whose offending peaked in their mid-20’s, medium rate offenders who continued to offend, offenders who continued to offend over time at a high rate and infrequent offenders who began offending in adulthood (see Fig. 2).

A series of binary logistic regression models were fitted to analyse the relationship between gambling cluster and criminal career. Firstly, it should be noted that no association was found between the PGSI categories of low risk, medium risk and problem gambler with criminal career. Secondly, no association was found between severe gambling harm, as identified in our LCA model (clusters five and six) and criminal career. This part of the analysis confirmed that all types of offenders can experience severe gambling harm. However, a significant association was found between higher level loss chasing (cluster three) and high rate offending in criminal careers. High rate mid 20’s peak offenders are 5.3 times more likely and high rate chronic persisters are 3.7 times more likely than others to be more frequent loss chasers who claim to be sometimes affected by the consequences of their gambling behaviour. 9.3 % of the PNC sample fell into this group, over half of whom met the PGSI threshold for problem gambling.

### Gambling Harm and Drug/Alcohol Use

Several studies have found correlations between harmful gambling behaviour and substance use (Cowlishaw et al. 2014) and also between problem gambling and being charged with drug offences (Laursen et al. 2016). To test whether substance use and gambling harm were co-symptomatic the relationship between the gambling behaviour clusters and substance use in the total prisoner cohort was also analysed. Substance use was highly prevalent in this prison population and it was possible to create the substance use variable for two thirds of the total respondents who gave valid answers to both questions used for the ‘Type of Drug User’ variable (n = 697). A Pearson Chi square test was carried out for all 697 respondents for whom we were able to create the complete data. This found no statistical association between the gambling behaviour sub-types and drug use in our sample (*p* .48) (Table 7).

**Table 7:** LCA gambling behaviour clusters and alcohol/drug use

Substance use	Latent gambling group						Total
	Abstainer non-problem gamblers	Occasional excitement chasers	Occasional loss-chasers (higher-level not affected)	Occasional loss-chasers (higher level affected)	Un-assertive non-problem gamblers	Problem gamblers	
Daily user not received help	75	16	11	9	4	2	117
Weekly/monthly user not received help	90	19	13	6	4	5	137
Daily user received help	253	41	37	21	4	2	358
Weekly/monthly user received help	57	10	12	3	2	1	85
Total	475	86	73	39	14	10	697

A bias was noted in this subset, with an under-representation of non-gamblers and an over-representation of the three types of ‘occasional loss chasers’. There was also an issue with small counts in some of the sub-categories in these data^1^ owing to the counts in the smaller gambling groups—‘unassertive non-problem gamblers’ and the ‘problem gamblers’—in each of the drug using types. However, when a Chi square test was carried out on the larger groups, excluding ‘unassertive non-problem gamblers’ and the ‘problem gamblers’, the associated *p value* was .88. We can therefore be confident in accepting the null hypothesis that gambling behaviour and drug and/or alcohol use are not co-symptomatic in this sample (see Table 7).

However, when we examine the types of substances that the prisoners have used within the past 12 months some interesting patterns emerge. The three occasional gambling groups are less likely to have abstained from alcohol and drug use compared to other groups (Table 8). Furthermore, nearly two thirds of the problem gamblers have abstained from any type of substance use. And compared to all other groups except the unassertive gamblers, the problem gamblers are much less likely to have used suppressants and stimulants and none have used hallucigens and opiates. This suggests that problem gamblers get their needs met primarily from gambling and not from drugs or alcohol. This finding appears to contrast with the finding in Table 5 that the most common type of crime that led to imprisonment for the problem gamblers are drug offences. However, it is important to note that, firstly numbers of problem gamblers included in Table 5 are small and only four offenders received such convictions, and secondly these four convictions were all specifically for possession with intent to supply. It is possible that some of these offenders are suppliers, not users. It is clear nevertheless that further exploration into differences between types of gambler and their (poly)substance use would provide important insights into the nature, if indeed any exists, of the co-symptomatic relationship between substance use and gambling behaviour. To do so in this paper, though, would detract from our main focus.

**Table 8:** Prevalence of drug use in last year by latent gambling group

Latent gambling group	Nothing	Alcohol	Opiates	Suppressants	Stimulants	Hallucinogens	Other
Abstainers/non-problem gamblers (n = 750)	56.5	27.3	22.5	27.4	15.2	3.5	8.0
Occasional excitement chasers (n = 111)	40.5	43.2	27.0	40.5	30.6	5.4	14.4
Occasional loss-chasers (higher-level less affected) (n = 98)	45.9	40.8	22.4	35.7	24.5	6.1	10.2
Occasional loss-chasers (higher level affected) (n = 52)	38.5	46.2	28.8	44.2	30.8	11.5	21.2
Unassertive gamblers (n = 24)	75.0	16.7	8.3	12.5	4.2	0.0	0.0
Gamblers affected by high level of problems (n = 21)	61.9	38.1	0.0	9.5	9.5	0.0	4.8

## Discussion

Several links between gambling and crime have been previously proposed including; crime committed to fund gambling, the co-occurrence of addiction and associated criminal behaviour and the role of gambling as part of the social milieu of an illegal lifestyle (Campbell and Marshall 2007). The current analysis adopted criminal career theory (Soothill et al. 2009), which examines patterns of offending across the life-course, to test whether a ‘co-symptomatic’ relationship (Lahn and Grabosky 2003) exists between different criminal careers and gambling behaviours. For example, is gambling harm associated with long-term chronic and persistent criminal careers or those that peak in adolescence and young adulthood? Data on gambling prevalence suggest that the latter would contain higher levels of problem gambling since younger age groups appear more vulnerable (Wardle et al. 2011). However, the application of criminal career analysis to this sample of prisoners reinforces that links between gambling and crime remain complex. Three findings are worthy of note; firstly, the study confirms that gambling harm continues to be significantly higher in incarcerated populations than in the general population. Secondly, high rate offenders (both chronic offenders and those peaking in young adulthood) are also significantly more likely to be frequent loss chasers experiencing some level of harm as a consequence. Thirdly, those prisoners with the most severe problems with gambling feature across all types of criminal career.

Three sub-types, or pathways, of problem gambling that are not mutually exclusive were proposed by Blaszczynski and Nower (2002); behaviourally conditioned or ‘otherwise normal’ problem gamblers, ‘anti-social’ impulsivity problem gamblers and emotionally vulnerable problem gamblers. Subsequent studies have aimed at finding empirical support for the pathways theory with some success (Turner et al. 2008; Milosevic and Ledgerwood 2010). Offending is de facto ‘anti-social behaviour’ and as such our whole sample might be said to be ‘anti-social’ to some degree. The ‘antisocial impulsivity’ problem gambler is characterized by early age onset of gambling and early entry into offences related to gambling, suggesting a further relationship to offending populations and thus also a prison population with repeated offences across their criminal career, unless some form of intervention curtails the behaviour. The finding of an association between high rate offenders whose criminal career begins from the age of 10, and more frequent loss chasing in this group would support the theory that these prisoners (9.3 %) are on an ‘antisocial impulsivity’ pathway.

Emotionally vulnerable problem gamblers are notable for high levels of depression, anxiety and substance use in addition to their gambling behaviour. Given the repeated findings from research concerning the co-occurrence of substance use and crime (Chandler et al. 2009; UKDPC 2008) it may be anticipated that substance use would also be evident in the present sample. This was found to be the case with 65.9 % of the total sample found to be daily, weekly or monthly drug and/or alcohol users, over half of whom had received help for their substance use. There is a growing literature on the co-occurrence of substance use and problem gambling. For example, a meta-review covering studies between 1998 and 2010 found a mean prevalence rate for substance use co-morbidity and problem gambling in general population studies of 57.5 % (Lorains et al. 2011). In support of addiction theories of problem gambling, the most recent version (DSM-5 2013) classifies gambling disorder with substance use disorders as a behavioural addiction for the first time (DSM-5 2013). However, the present analysis could find no significant association between substance use and gambling behavior in this prison population. Whilst the majority of the sample met the substance use criteria, 68 % of substance using prisoners either did not gamble or gambled without experiencing problems, with the remaining 32 % experiencing some negative effects from their gambling compared with 29 % across the whole sample.

Rather than attributing links between gambling and crime to specific crimes such as fraud, theft and financial crimes, this data is suggestive of a potential ‘co-symptomatic’ connection between gambling and crime, in supporting the theory that both may be connected by impulse control. Gambling has long been associated with a heightened need for excitement, or ‘action’ seeking (Lesieur 2001) and impulsivity (Blaszczynski and Nower 2002; Grant and Potenza 2012). Up until 2013 pathological gambling was defined as an impulse control disorder (APA 1994). Similarly, criminal behaviour has been associated with impulse control disorders (Farrington 1995; Gottfredson and Hirschi 1990; Grant and Potenza, 2012). Blaszczynski and Nower’s (2002) pathways model of gambling acknowledges heterogeneity and the inadequacy of any single theoretical explanation. The present study did not test directly for impulsivity but within the PGSI three items might be associated with impaired control: going back to try to win money lost (loss-chasing), betting more than one could afford to lose and gambling more to get the same amount of excitement. The latent cluster analysis supports the prevalence of loss chasing and/or ‘excitement chasing’, and thus impaired control, as a defining feature for 92 % of those who experience *any* adverse consequences at lower or higher levels (Clusters 2, 3, 4 and 6 above). Further research on the relationship between gambling and crime careers and impulsivity would be required to test this hypothesis.

Findings regarding gambling behavior and consequences in this study have implications for treatment options in prison. The treatment literature in relation to gambling behaviour sub-types is equivocal. For example, Nower and Blaszczynski (2005) propose that youth impulsivity pathway gamblers are more resistant to treatment, whilst Ledgerwood and Petry (2010) found that sub-typing did not predict treatment outcomes. Given that 43 % of PGSI problem gamblers in the present study were defined almost entirely by their level of loss chasing this could offer some explanation: if they are ‘impulsivity pathway gamblers’ and if it is perceived that adverse consequences rarely ensue from this behaviour the motivation to engage with treatment interventions will be reduced. Gamblers in these groups may be unlikely to self-identify as having a problem with gambling at all. Furthermore, motivation to engage with treatment is a complex issue in prison settings in that prisoners may be reluctant to admit to undiagnosed problems that might impact on their release dates. The occasional loss chasers, who comprise the majority (68.1 %) of gamblers in our sample, may be appropriate targets for interventions that raise awareness of the harm potential of gambling. Education and treatment interventions could recognise high rate offenders as a vulnerable group to target for gambling harm prevention. Interventions that focus on impulse control as co-symptomatic, combining offending and gambling impulses, may prove beneficial with this group.

Limitations should be noted. Data on gambling careers was reliant on prisoners consenting to access the UK Police National Computer and submitting fully completed responses to the PGSI. Only 41.3 % o (437) of the total sample met both criteria which means that statistical analysis of the smaller sub-groups should be treated with caution. Although it is a well validated and supported measure there are also ongoing debates about the cut off points for the PGSI (Stone et al. 2015) and follow up for false positives and false negative scores were not part of the present study.

## Conclusion

This research confirms international findings of significantly higher levels of problem gambling in offending populations compared to general population prevalence. We propose a more nuanced understanding of gambling harm can be gained through latent class analysis. Six different clusters emerged, three distinguished primarily by levels of loss chasing behaviour and its consequences. Furthermore, analysis of the offending trajectories of prisoners finds that gambling behaviour and crime may be indirectly connected through impulse control, particularly in relation to high rate offending crime careers. Gambling education and awareness programmes should aim to prevent future gambling harm for the lower level affected loss chasers, comprising approximately one fifth of those in the prison population in the UK, amounting to over 18,000 prisoners at current incarceration rates (Ministry of Justice 2015; Scottish Prison Service 2015). Many of these prisoners are unlikely to recognise that they may be negatively impacted by their gambling behaviour. High level affected loss chasers and serious problem gamblers who score highly on most elements of the PGSI (clusters 4 (4.9 %) and 6 (1.9 %) amounting to 6.8 % of the total) are likely to need more intensive therapeutic interventions. This would mean approximately 6000 prisoners require urgent targeted help aimed at reducing both the frequency of gambling behaviour and the serious harms they agree they are experiencing.

## Footnotes

1 At most 20 % of the expected frequencies should be less than 5. When all the data are analysed about 30 % (7) of the cells have *expected* frequencies that are less than 5.
